# Synaptic Organization of Perisomatic GABAergic Inputs onto the Principal Cells of the Mouse Basolateral Amygdala

**DOI:** 10.3389/fnana.2016.00020

**Published:** 2016-03-07

**Authors:** Viktória K. Vereczki, Judit M. Veres, Kinga Müller, Gergö A. Nagy, Bence Rácz, Boglárka Barsy, Norbert Hájos

**Affiliations:** ^1^Lendület' Laboratory of Network Neurophysiology, Institute of Experimental Medicine, Hungarian Academy of SciencesBudapest, Hungary; ^2^Department of Anatomy, Histology and Embryology, Faculty of Medicine, Semmelweis UniversityBudapest, Hungary; ^3^János Szentágothai School of Neurosciences, Semmelweis UniversityBudapest, Hungary; ^4^Department of Anatomy and Histology, Szent István UniversityBudapest, Hungary; ^5^Electronmicroscopy Research Group, Faculty of Veterinary Science, Szent István UniversityBudapest, Hungary

**Keywords:** network, inhibition, interneuron, synapse, GABA

## Abstract

Spike generation is most effectively controlled by inhibitory inputs that target the perisomatic region of neurons. Despite the critical importance of this functional domain, very little is known about the organization of the GABAergic inputs contacting the perisomatic region of principal cells (PCs) in the basolateral amygdala. Using immunocytochemistry combined with *in vitro* single-cell labeling we determined the number and sources of GABAergic inputs of PCs at light and electron microscopic levels in mice. We found that the soma and proximal dendrites of PCs were innervated primarily by two neurochemically distinct basket cell types expressing parvalbumin (PVBC) or cholecystokinin and CB_1_ cannabinoid receptors (CCK/CB_1_BC). The innervation of the initial segment of PC axons was found to be parceled out by PVBCs and axo-axonic cells (AAC), as the majority of GABAergic inputs onto the region nearest to the soma (between 0 and 10 μm) originated from PVBCs, while the largest portion of the axon initial segment was innervated by AACs. Detailed morphological investigations revealed that the three perisomatic region-targeting interneuron types significantly differed in dendritic and axonal arborization properties. We found that, although individual PVBCs targeted PCs via more terminals than CCK/CB_1_BCs, similar numbers (15–17) of the two BC types converge onto single PCs, whereas fewer (6–7) AACs innervate the axon initial segment of single PCs. Furthermore, we estimated that a PVBC and a CCK/CB_1_BC may target 800–900 and 700–800 PCs, respectively, while an AAC can innervate 600–650 PCs. Thus, BCs and AACs innervate ~10 and 20% of PC population, respectively, within their axonal cloud. Our results collectively suggest, that these interneuron types may be differently affiliated within the local amygdalar microcircuits in order to fulfill specific functions in network operation during various brain states.

## Introduction

Although a wealth of data indicates a critical role for the basolateral amygdala (BLA) in higher order cognitive functions, such as the control of memory formation, decision making, emotional reactions, or threat processing (Fanselow and Gale, 2003; Paré, 2003), the cell types and their connectivity within these neural networks are poorly understood. As in other cortical regions, the BLA is composed of glutamatergic principal cells (PCs) and a variety of GABAergic interneurons (McDonald, 1992; Sah et al., 2003; Pape and Pare, 2010). PCs comprise the vast majority of neurons in the BLA (80%) and provide the excitatory output of this structure by projecting to cortical and subcortical areas. To fulfill the physiological operation of amygdalar PCs during cognitive tasks, their firing should be precisely controlled, e.g., by local GABAergic interneurons. Even though this latter cell type is small in number (McDonald and Augustine, 1993), its impact on behavioral output is significant (Wolff et al., 2014), highlighting their role in proper PC operation and amygdalar function.

Previous studies have recognized that several GABAergic cell types are present in the BLA (Ehrlich et al., 2009; Spampanato et al., 2011), however, detailed comparative analysis of the morphological properties of these neurons is still obscure. A more complete knowledge of GABAergic interneurons in the BLA could help us to understand the function of these cells in physiological network operation. As GABAergic inhibition in the amygdala has been shown to be impaired in psychiatric disorders such as anxiety (Lüthi and Lüscher, 2014; Prager et al., 2014), uncovering the organization of synaptic inhibition may help to reveal the pathological mechanisms underlying diseases involving amygdalar malfunction.

One of the functional GABAergic cell groups in cortical areas preferentially innervates the perisomatic region of PCs (Miles et al., 1996; Freund and Katona, 2007). This region of PCs has been proposed to be a critical subcellular domain, because here the proximity to the site of the action potential generation allows the opening and closing of synaptic conductances to most effectively control the spiking of neurons (Spruston, 2008). The perisomatic region collectively refers to the axon initial segment (AIS), the soma, and the proximal dendrites, but the extent of the latter compartment has been only vaguely defined. Previous work revealed, that the perisomatic region of PCs in the BLA is exclusively innervated by GABAergic boutons, which were immunoreactive for type 1 cannabinoid receptors (CB_1_), or the Ca^2+^ binding proteins parvalbumin (PV), and/or calbindin (Calb) (Katona et al., 2001; McDonald and Betette, 2001; McDonald et al., 2002; Muller et al., 2006). However, neither the ratio of these GABAergic inputs nor the morphological features of the interneurons innervating the perisomatic region are known. As previous studies have shown, these GABAergic interneurons play a crucial role in several types of memory processes, including spatial working memory or fear memory acquisition (Fuchs et al., 2007; Murray et al., 2011; Wolff et al., 2014). In addition to the perisomatic region-targeting interneurons, another functional group of GABAergic cells innervating predominantly the dendrites of PCs has been identified in the amygdala. Among these cells subgroups can be differentiated based on the expression of neuropeptides (e.g., somatostatin, neuropeptide Y) or Calb (Muller et al., 2007; Bienvenu et al., 2012; Manko et al., 2012). In other cortical regions, a third functional group of GABAergic cells innervating selectively or preferentially other interneurons has been described (Acsády et al., 1996b; Gulyás et al., 1996), although their presence in the BLA is still a question. This specific class of GABAergic cells expresses vasoactive intestinal polypeptide and/or calretinin in the hippocampus or neocortex (Acsády et al., 1996a; Gulyás et al., 1996; Hajos et al., 1996; Meskenaite, 1997), neurochemical markers, which have been detected in a subpopulation of amygdalar GABAergic cells (McDonald, 1989, 1994). The comprehensive analysis of the characteristics of amygdalar interneurons could help us to understand the mechanisms that underlie associative learning related to the BLA.

Here, we first defined the extent of proximal dendrites that should be considered as a part of the perisomatic region of PCs in the BLA. Next, we determined the number and source of GABAergic inputs covering the soma and proximal dendrites of PCs. Finally, we characterized the morphological properties of the interneurons giving rise to the vast majority of synaptic inputs onto the perisomatic region of PCs. We found that three distinct types of GABAergic cells with characteristic neurochemical features provide synaptic inputs with different properties onto the perisomatic region of PCs, which may ensure a complex regulation of PC spiking during distinct brain states.

## Materials and methods

All experiments were approved by the Committee of the Scientific Ethics of Animal Research (22.1/4027/003/2009) and were performed according to institutional guidelines of ethical code and the Hungarian Act of Animal Care and Experimentation (1998. XXVIII. Section 243/1998, renewed in 40/2013) in accordance with the European Directive 86/609/CEE and modified according to the Directives 2010/63/EU.

### Principal cell reconstructions and analysis of the inputs onto their perisomatic region *in vitro*

#### Labeling of neurons in slice preparations

*In vitro* biocytin labeling of neurons was carried out as described before (Veres et al., 2014). Briefly, three P18-22 CD1 mice were deeply anesthetized with isoflurane and decapitated. The brain was quickly removed and placed into ice-cold cutting solution containing (in mM): 252 sucrose, 2.5 KCl, 26 NaHCO_3_, 1 CaCl_2_, 5 MgCl_2_, 1.25 NaH_2_PO_4_, 10 glucose, bubbled with 95% O_2_/5% CO_2_ (carbogen gas). Horizontal slices of 200 or 300 μm thickness (interaural plane 0.9–1.4 mm) containing the amygdala region were prepared with a Leica VT1000S or VT1200S Vibratome (Wetzlar, Germany), and kept in an interface-type holding chamber containing artificial cerebrospinal fluid (ACSF) at 36°C that gradually cooled down to room temperature. ACSF contained (in mM) 126 NaCl, 2.5 KCl, 1.25 NaH_2_PO4, 2 MgCl_2_, 2 CaCl_2_, 26 NaHCO_3_, and 10 glucose, bubbled with carbogen gas. Neurons were selected randomly to give the highest probability to sample all morphological types of PCs in the BLA. Targeted cells were recorded under visual guidance using differential interference contrast microscopy (Olympus BX61W) and laid 50–100 μm below the surface of the slice. PCs were recorded in whole-cell mode using a K-gluconate based intrapipette solution containing biocytin to label their dendritic and axonal arbor [intrapipette solution (in mM): 110 K-gluconate, 4 NaCl, 2 Mg-ATP, 20 HEPES, 0.1 EGTA, 0.3 GTP (sodium salt), 10 phosphocreatine, and 0.2% biocytin adjusted to pH 7.3 using KOH and with an osmolarity of 290 mOsm/L]. After fixation in 4% paraformaldehyde (PFA), Alexa 647-coupled streptavidin (1:2000, Invitrogen) was used to visualize the fine details of the neurons in the entire slice.

#### Visualization of boutons closely opposing the perisomatic region of *in vitro* labeled cells

To quantify excitatory inputs, immunostainings were carried out using rabbit anti-VGLUT1, guinea pig anti-VGLUT2, and mouse anti-bassoon primary antibodies visualized by donkey anti-rabbit IgG coupled with DyLight 405, donkey anti-guinea pig-Alexa 488, and donkey anti-mouse-Cy3 secondary antibodies (all 1:500, Jackson ImmunoResearch Laboratories Inc., West Grove, PA). To assess the GABAergic inputs of these cells, we carried out immunostainings using guinea pig anti-VGAT and goat anti-panGAD primary antibodies, visualized by Cy3 (donkey anti-guinea-pig and donkey anti-goat, 1:200, Jackson ImmunoResearch Laboratories Inc.). To label Kv2.1 we used mouse anti-Kv2.1, which was visualized with an Alexa 488-conjugated donkey anti-mouse antibody. Slices were mounted in Vectashield (Vector Laboratories, Burlingame, CA), and high resolution images were taken with an A1R confocal laser scanning microscope (0.06 μm/pixel, 0.13 μm z-step size; CFI Plan Apo VC 60X Oil N.A. 1.4 objective, Nikon Europe, Amsterdam, The Netherlands). Using the 3D confocal images taken from the labeled cells, the soma surface of recorded cells and their dendritic trees decorated with spines were reconstructed with Neurolucida 10.53 software. The putative inputs onto the cells were reconstructed by labeling the sites where the presynaptic boutons (i.e., VGLUT1 or VGLUT2 together with bassoon or panGAD/VGAT-containing profiles) closely opposed the soma or dendrite of the biocytin-filled cell. The extent of the Kv2.1 staining on the dendrites of biocytin-filled PCs was also marked. In the same material, GABAergic inputs of neighboring cells immunostained for Kv2.1 were also reconstructed. Values were corrected for shrinkage and flattening of the tissue (x,y: no correction, z:1.7). Shrinkage correction factors in the x and y axes were calculated by comparing the distance between characteristic landmarks in the slice (e.g., external capsule, anterior commissure, optic tract, alveus) immediately after slice preparation and after mounting the slices (*n* = 10) using an epifluorescent microscope (Zeiss AxioImager Z1). Z axis shrinkage correction factor was calculated by comparing the vibratome-cut slice thickness and the thickness measured with a confocal microscope (A1R, Nikon) after mounting. A summary of all the primary antibodies used can be found in Table 1.

**Table 1:** **Summary of the primary antibodies used in the study**.

**Molecule**	**Species**	**Provider**	**Code**	**Specificity**	**Dilution**	**Figures**
VGLUT1	Rabbit	SYSY	135302	Todd et al., 2003 and manufacturer information	1:10.000	
VGLUT2	Guinea pig	SYSY	135404	Manufacturer information and labeling pattern as published with other antibodies.	1:1000	
Bassoon	Mouse	Abcam	SAP7F407	Ripley et al., 2011 and manufacturer information	1:3000	
VGAT	Guinea pig	Frontier Inst.	VGAT-GP-Af1000	Manufacturer information	1:1000	Figures 1A, 2
pan-GAD	goat	Frontier Inst.	GAD-Go-Af240	Veres et al., 2014 and manufacturer information	1:500	Figure 1A
Kv2.1	mouse	Neuromab	75-014	Trimmer, 1991 and manufacturer information	1:1000	Figures 1E, 2A,C, 3
CB_1_	Rabbit	Cayman	10006590	Manufacturer information	1:1000	Figure 2A
CB_1_	Goat	Frontier Inst.	CB1-Go-Af450	Fukudome et al., 2004 and manufacturer information	1:1000	Figures 2C, 3
CB_1_	Rabbit	Ken Mackie	Gift	Hájos et al., 2000	1:500	
PV	goat	Swant	PVG-214	Manufacturer information and labeling pattern as published with other antibodies	1:5000	Figure 2A
PV	Guinea pig	Sysy	195,004	Bienvenu et al., 2012, manufacturer information and labeling pattern as published with other antibodies.	1:10.000	Figure 2C
PV	rabbit	Swant	PV 25	Manufacturer information and labeling pattern as published with other antibodies	1:5000	
Calb	Rabbit	Swant	CB-38a	Airaksinen et al., 1997 and manufacturer informations.	1:5000	Figures 2C, 3, 4
Calb	Guinea pig	Sysy	214 004	Manufacturer information	1:5000	
Ankyrin G	Rabbit	Santa Cruz	sc-28561	Manufacturer informations and labels axon initial segments as published with other antibodies.	1:200	
Ankyrin G	Mouse	Santa Cruz	sc-12719	Manufacturer informations and labels axon initial segments as published with other antibodies.	1:200	Figures 3B, 4
GFP	Goat	Abcam	ab5450	Manufacturer information	1:5000	
CCK	Mouse	CURE, UCLA	9303	Manufacturer information	1:2000	
CCK	Rabbit	Sigma Aldrich	C2581	Manufacturer information	1:8000	

### Evaluation of GABAergic inputs on the perisomatic region of principal cells *in vivo*

#### Preparation of tissue samples from mouse brain

C57Bl/6J mice were deeply anesthetized and transcardially perfused with either 2.5% acrolein in 4% PFA (pH 6.8) in 0.1 M phosphate buffer (PB) for 10 min followed by post-perfusion fixation in 4% PFA in 0.1 M PB for 1.5 h (3 mice) or with 2% PFA in 0.2 M Na-acetate buffer (pH 6.0) for 20 min without post-fixation (2 mice). For all cases, the part of the brain containing the amygdala was sectioned into 40 μm-thick sections, which were soaked in 30% sucrose overnight and the sections were kept in cryoprotectant anti-freeze solution consisting of sucrose, ethylene glycol, distilled H_2_O, and phosphate-buffered saline (3:3:3.1 volume ratio) at –20°C until further processing was initiated. Prior to immunostaining the cryoprotectant was washed out in 0.1 M PB. Brains of three additional C57Bl/6J mice were quickly removed from the skull under deep anesthesia without transcardial perfusion, and a block containing the amygdala region was dissected followed by immersion into a fixative containing 2% PFA in 0.1 M PB for 2 h. After fixation the blocks were rinsed several times in 0.1 M PB and re-sectioned to 40 μm thickness.

#### Visualization of boutons opposing the perisomatic region of neurons

For the analysis of the GABAergic inputs onto the soma and proximal dendrites, the sections were blocked in 10% Normal Donkey Serum (NDS, Vector Laboratories) in Tris-buffered saline (TBS, pH 7.4) followed by incubation in a mixture of primary antisera of rabbit anti-CB_1_, guinea pig anti-VGAT, goat anti-PV and mouse anti-Kv2.1 diluted in TBS containing 1% NDS and 0.1% Triton X-100 for 3 days at 4°C. Following several washes in TBS, the sections were incubated in a mixture of secondary antisera of DyLight 405-conjugated donkey anti-rabbit, Alexa 488-conjugated donkey anti-guinea pig, Cy3-conjugated donkey anti-mouse, and Alexa 647-conjugated donkey anti-goat (all 1:500, Jackson ImmunoResearch Laboratories Inc.). To determine the Calb content of PV- and CB_1_-immunoreactive varicosities at the perisomatic region, sections were incubated in a mixture of primary antisera of guinea pig anti-CB_1_, rabbit anti-Calb and mouse anti-Kv2.1 or in a mixture of antisera of guinea pig anti-PV, rabbit anti-Calb and mouse anti-Kv2.1 diluted in TBS containing 1% NDS and 0.1% Triton X-100 for 3 days at 4°C. Following several washes in TBS, the sections were incubated in a mixture of secondary antisera of Alexa 488-conjugated donkey anti-rabbit, Alexa 647-conjugated donkey anti-guinea pig and Cy3-conjugated donkey anti-mouse (all 1:500, Jackson ImmunoResearch Laboratories Inc.) diluted in TBS for 2 h. Following several washes in TBS, sections were mounted on glass slides in Vectashield (Vector Laboratories). For quantitative analysis, high resolution (60 nm/pixel) z-stack images (z-step size: 130 nm) were taken from the upper 5 to 10 μm of the slices using an A1R confocal laser scanning microscope (objective: CFI Plan Apo VC 60X Oil N.A. 1.4).

#### Visualization of calb-expressing boutons contacting the AISs

For the evaluation of Calb inputs onto the AISs the sections were incubated in 10% NDS containing 0.05% Triton-X 100 in 0.1 M PB for 45 min followed by the incubation in a mixture of antisera mouse anti-ankyrin G IgG to visualize AISs and rabbit anti-Calb at 4°C for 3 days. The primary antibodies were visualized by the incubation of Cy3-conjugated donkey anti-mouse IgG and Alexa 647-conjugated donkey anti-rabbit IgG for 2 h at room temperature (all 1:500, Jackson ImmunoResearch Laboratories Inc.). In a separate experiment, sections were incubated in a mixture of antisera; mouse anti-ankyrin G and rabbit anti-CB_1_. These primary antisera were visualized with Cy3-conjugated donkey anti-mouse and Alexa 488-conjugated donkey anti-rabbit secondary antibodies (1:500, Jackson ImmunoResearch Laboratories Inc.). In both cases, sections were washed and mounted on the slides in Vectashield. Confocal images were collected using a Nikon A1R microscope (z step size: 0.13 μm, xy: 0.06 μm/pixel). Based on the 3D confocal image, the AISs of cells were delineated with the Neurolucida 10.53 software and the sites where the presynaptic boutons (i.e., Calb/CB_1_-containing profiles) made close apposition with ankyrin G-immunostained profiles were labeled as contact sites along the AISs. For the analysis of the obtained data the Neurolucida Explorer software was used. Values were corrected for shrinkage of the tissue (x, y, z axis correction: 1.08).

### Neurochemical marker detection in *in vivo* labeled interneurons

#### Evaluation of neurochemical content of interneurons expressing fluorescent proteins in transgenic mice

Transgenic mice expressing either enhanced green fluorescent protein (eGFP) controlled by parvalbumin (PV) promoter (BAC-PV-eGFP) (Meyer et al., 2002) or red fluorescent protein under the control of cholecystokinin (CCK) promoter were used (BAC-CCK-DsRed; Máté et al., 2013) for interneuron labeling. To confirm previous findings obtained in the BLA of the PV-eGFP strain (Meyer et al., 2002), we first performed double immunofluorescent labeling using antibodies against PV (rabbit anti-PV) and eGFP (goat anti-GFP). We found that the somata of the vast majority of the eGFP-labeled neurons (93.5%) were also PV immunoreactive (274 out of 293 cells; *n* = 3 animals). Similar investigations were performed in slices prepared from BAC-CCK-DsRed mice using a mixture of antibodies developed against CCK (mouse anti-CCK) and CB_1_ (rabbit anti-CB_1_, 1:500, Bodor et al., 2005). Cells with the highest level of fluorescent signal were sampled for recordings, because most of the cell somata with intense fluorescent signal were colocalized with CCK (96.6%, 114 out of 118; *n* = 3 mice) or triple-labeled for CCK and CB_1_ (90.7%, 107/118; *n* = 3 mice). Besides this good correspondence, the CB_1_ immunoreactivity at the boutons of each biocytin-filled interneurons recorded in BAC-CCK-DsRed mouse slices was evaluated *post-hoc* using immunofluorescent staining (rabbit anti-CB1), and only immunopositive interneurons were included in this study.

#### Labeling of interneurons in slice preparations prepared from transgenic mice and determination the neurochemical content of biocytin-filled cells

Slices were prepared as described above for labeling PCs. EGFP or DsRed in cells was excited by a UV lamp, and the fluorescence was visualized by a CCD camera (Hamamatsu Photonics, Japan). Cells were filled with the same K-gluconate based intrapipette solution as used for PC labeling containing biocytin, and labeled interneurons were visualized by Alexa 488- or Cy3-coupled streptavidin (1:3000, Invitrogen or Sigma-Aldrich, respectively). In those cases when the Calb content of PV-expressing interneurons was evaluated, the sections were first incubated in guinea pig anti-Calb primary antibody diluted in 0.1 M PB containing 1% NDS and 0.1% Triton X-100 for 3 days at 4°C. Then the sections were incubated in DyLight 649-conjugated donkey anti-guinea pig secondary antibody (1:300, Millipore) diluted in 0.1 M PB for 2 h. The Calb content of PV-containing interneurons was investigated in the varicosities and, in most cases, in the soma and dendrites of biocytin-filled cells using confocal microscopy. To determine the CB_1_ content of interneurons sampled in slices prepared from CCK-DsRed mice, slices were re-sectioned to 40 μm thickness using a vibratome and first blocked in Normal Goat Serum (NGS, 10%, Vector Laboratories) diluted in TBS (pH 7.4) containing 0.1 % Triton X-100 followed by incubation in rabbit anti-CB_1_ in TBS containing 2% NGS and 0.1% Triton X-100. Following several washes in TBS, CB_1_ expression was visualized using DyLight 405 (goat anti-rabbit, 1:500, Jackson ImmunoResearch Laboratories Inc.). The CB_1_ content of recorded interneurons was investigated in the varicosities of biocytin-filled cells.

### Anatomical identification of biocytin-filled interneurons

After recordings, the slices were fixed in 4% PFA in 0.1 M PB (pH 7.4) overnight, followed by washout with 0.1 M PB several times. Biocytin-filled cells from PV-eGFP mice were visualized with Alexa 488-conjugated streptavidin (1:3000, Invitrogen), while filled interneurons recorded in slices prepared from CCK-DsRed mice were visualized with Cy3-conjugated streptavidin (1:1000; Sigma-Aldrich). If the visualization of biocytin in the axon collaterals of filled neurons was successful, then slices were embedded in agar (1%) and re-sectioned to 40 μm thickness. In each case, when interneurons were recorded in slices prepared from PV-eGFP mice, we separated axo-axonic cells (AAC) from basket cells using double immunofluorescent staining for biocytin and rabbit anti-ankyrin G to visualize the biocytin-filled axon collaterals together with the AIS of neurons. When boutons of a labeled interneuron formed many close appositions with ankyrin G-immunostained profiles, the cell was identified as an axo-axonic cell (see for details, Gulyás et al., 2010; Veres et al., 2014).

### Target distribution analysis of biocytin-filled interneurons

To estimate the target distribution of PVBCs using light microscopy, sections were incubated in mouse anti-Kv2.1, which was visualized with Cy3-conjugated donkey anti-mouse antibody (1:200, Jackson ImmunoResearch Laboratories Inc.). In the case of CCK/CB_1_-expressing BCs (CCK/CB_1_BCs), sections were incubated in a mixture of mouse anti-Kv2.1 and rabbit anti-CB_1_ primary antibodies visualized with Alexa 488 (donkey anti-mouse, 1:200, Molecular Probes) and DyLight 405 (donkey anti-rabbit, 1:200, Jackson ImmunoResearch Laboratories Inc.). Following several washes in TBS, sections were mounted on glass slides in Vectashield (Vector Laboratories). Images were taken using an A1R or a C2 confocal laser scanning microscope (Nikon Europe, Amsterdam, The Netherlands) using a 60 × (NA = 1.4) apochromatic objective (z step size: 0.13 μm, xy: 0.06 μm/pixel).

### Interneuron reconstructions and electron microscopic analysis of the postsynaptic targets of interneurons

For the reconstruction of the axonal and dendritic tree of interneurons, cells were labeled in 300-μm-thick horizontal amygdalar slices as described above for labeling PCs. After biocytin filling, slices were fixed in a solution containing 4% PFA, 0.05% glutaraldehyde, and 15% picric acid in 0.1 M PB (pH 7.4) overnight. Slices were then washed out with PB several times, and incubated in cryoprotective solution (30% sucrose in 0.1 M PB) for 2 h, and then freeze-thawed three times above liquid nitrogen. Biocytin in PV-expressing cells was visualized using avidin-biotinylated horseradish peroxidase complex reaction (ABC; Vector Laboratories) with nickel-intensified 3,3-diaminobenzidine (DAB-Ni) producing a dark brown reaction product. In case of CCK-containing cells, first the CB_1_ expression of biocytin-filled axons was evaluated with fluorescent immunolabeling (see above). If the cell was proven to be immunopositive, slices were incubated in ABC, and the biocytin content of interneurons was developed by peroxidase reaction as described for PV cells. For interneuron reconstruction we selected those cells that were strongly and reliably labeled with biocytin (i.e., their processes ended within the slices or were cut at the surface of the slices, but the labeling did not fade with distance). Slices re-sectioned to a thickness of 60 μm were then postfixed in 0.5% OsO_4_, treated in 10% uranyl acetate, dehydrated in a graded series of ethanol followed by acetonitrile treatment, and embedded in epoxy resin (Durcupan; Sigma). Axonal and dendritic processes of the DAB-Ni visualized cells in 60-μm-thick sections were reconstructed with Neurolucida 10.53 software. Values were corrected for shrinkage and flattening of the tissue (x and y axis correction: 1.25, z axis correction: 2.5).

In 300 μm-thick *in vitro* slices we estimated that ~60–65% of the total arborization of individual interneurons could be recovered. This approximation is based on the findings that (1) after re-sectioning the slices, very few axon collaterals could be observed and few dendritic processes ended in the 60 μm-thick section furthest from the soma, implying that more than half of the neural processes should be present in the slices if we approximate a roughly spherical or cylindrical extension of interneurons in space, and (2) the soma of interneurons was sampled 50–100 μm deep from the slice surface.

Branched Structure, Convex Hull and Sholl Analyses were performed on the reconstructed interneurons with Neurolucida Explorer software. Based on the reconstruction of each cell, Branched Structure analysis provided information about the number and length of axons and dendrites by dendritic order and the number of branching points on axons and dendrites (referred to as nodes). The Convex Hull Analysis reports the area of a projection field. For Sholl analysis, concentric spheres at 50 μm radius intervals were drawn around the cell, centered on the cell body, and several parameters were measured independently for each shell (Table 2). The number of processes crossing each sphere border is referred to as the number of intersections.

**Table 2:** **Morphological parameters of the three perisomatic region-targeting interneurons in the BLA**.

	**PVBC**	**AAC**	**CCK/CB_1_BC**	**ANOVA**	**PVBC vs. AAC**	**PVBC vs. CCK/CB_1_BC**	**AAC vs. CCK/CB_1_BC**
n	7	7	5				
Dendritic length (μm)	7906.1 ± 2498.2	4606.1 ± 2645.7	9821 ± 2132.9	**0.007**	0.02	0.21	0.002
Dendritic tree/amygdala	0.119 ± 0.046	0.047 ± 0.022	0.101 ± 0.024	**0.005**	0.002	0.42	0.028
Dendritic surface × 10^3^ (μm^2^)	22.76 ± 8.11	11.88 ± 10.45	33.09 ± 7.49	**0.003**	0.037	0.06	<0.001
n	4	4	4				
Axon length × 10^4^ (μm)	7.816 ± 2.12	3.449 ± 2.421	6.1518 ± 1.134	**0.034**	0.012	0.26	0.08
Axon arbor/amygdala	0.356 ± 0.135	0.121 ± 0.037	0.251 ± 0.065	**0.015**	0.005	0.12	0.049
# of axon nodes	752 ± 245	426.5 ± 181.1	328.5 ± 92.3	**0.024**	0.033	0.009	0.47
# of varicosities	8369.5 ± 2550.1	3279.2 ± 773.2	4432.5 ± 1596.7	**0.007**	0.003	0.012	0.38
Inter-bouton distance (μm)	5 ± 0.55	4.35 ± 1.32	6.78 ± 1.59	*<**0.001***	*<0.001*	*<0.001*	*<0.001*

*Statistical comparisons were performed with ANOVA, except those shown in italic, which were obtained by Kruskal-Wallis ANOVA and Kolmogorov-Smirnov test*.

For electron microscopy, ultrathin sections of 60 nm thickness were cut, and following Reynold's lead citrate treatment, synaptic targets of biocytin-filled axon terminals were analyzed in serial sections using a Jeol electron microscope and the ImageJ software (JEM-1011, JEOL Ltd., Tokyo, Japan).

### Stereology

Materials included in quantitative analyses were taken from 3 mice. One hemisphere per animal and one horizontal block containing the entire amygdala was selected for quantitative analyses. Using a random starting point 40 μm-thick sections were stained with Nissl staining for estimating total number of neurons. Neurons were defined as large cells with identifiable, euchromatic nuclei and discrete, visible nucleoli, and some basophilic somatic staining could usually be observed (Vereczki et al., 2006). Compared to the neuronal nucleus, the nucleus of glial cells is dense, dot-like, heterochromatic, without visible nucleoli or somatic staining. Consequently, nuclear profiles were used as counting units.

Quantitative analyses were performed on a computer assisted image analysis system consisting of a Zeiss Axiophot microscope equipped with a MBF MS-88 computer-controlled motorized stage and using the StereoInvestigator program (MicroBrightField, Wiliston, VT). Tracings were made from the entire amygdala. The rationale of counting not only the number of neurons in the BLA but also in its neighboring areas was that the axonal cloud of interneurons was not restricted to the BLA, but could also occupy a part of the LA or BMA to a different degree. A total of six or seven tracings were made per amygdala, per hemisphere, in each animal. The traced sections were evenly spaced 240 μm apart. After outlining the boundary of amygdala at low magnification (5x) on the computer graphic display in each section separately, the software placed within each subfield boundary a set of optical disector frames (50 × 50 μm), in a systematic-random fashion. Neurons were then counted in optical disectors 9 μm in depth, according to stereologic principles (West et al., 1991; Nimchinsky et al., 2000). All analyses were performed using a 63x Plan-NeoFluar Zeiss objective (1.4 N.A.), auxiliary condenser lens (1.4 N.A) and Koehler illumination to achieve optimal optical sectioning during disector analysis. To ensure robustness of the data (Schmitz and Hof, 2000), we sampled 841 neurons in mouse #1, 875 in mouse #2 and 783 in mouse #3 (average: 833 neurons/mouse), and the coefficient of variation (CoV) calculated according to Schmitz and Hof (2000) were determined as 0.034 in mouse #1 0.034 in mouse #2 and 0.036 in mouse #3 (average: 0.035/mouse).

### Statistical analysis and distribution fitting

For comparison of data with a non-normal distribution according to the Shapiro-Wilk test, the Mann-Whitney U-test (M-W test) and Kruskal-Wallis ANOVA (K-W ANOVA), for data with normal distribution the two-sample *t*-test (*t-*test) and ANOVA were used. For the comparison of distributions, the two sample Kolmogorov-Smirnov test was used (K-S test). All statistics were performed using Origin 8.6 (Northampton, MA). Data are presented as mean ± SD unless indicated. For the analysis of spine and E/I ratio distribution a sigmoid curve (Boltzmann function) was fitted to the data with the following equation using Origin 8.6:

$$\begin{array}{l} y=\frac{{A_{1}\;-\;A}_{2}}{1+\;e^{\left(x\;-\;x_{0}\right)∕}dx}\;+\;A_{2} \end{array}$$

## Results

### The extent of the perisomatic region along the dendrites of principal cells in the BLA

#### Spine distribution along the proximal dendrites of PCs

**Figure 1:**
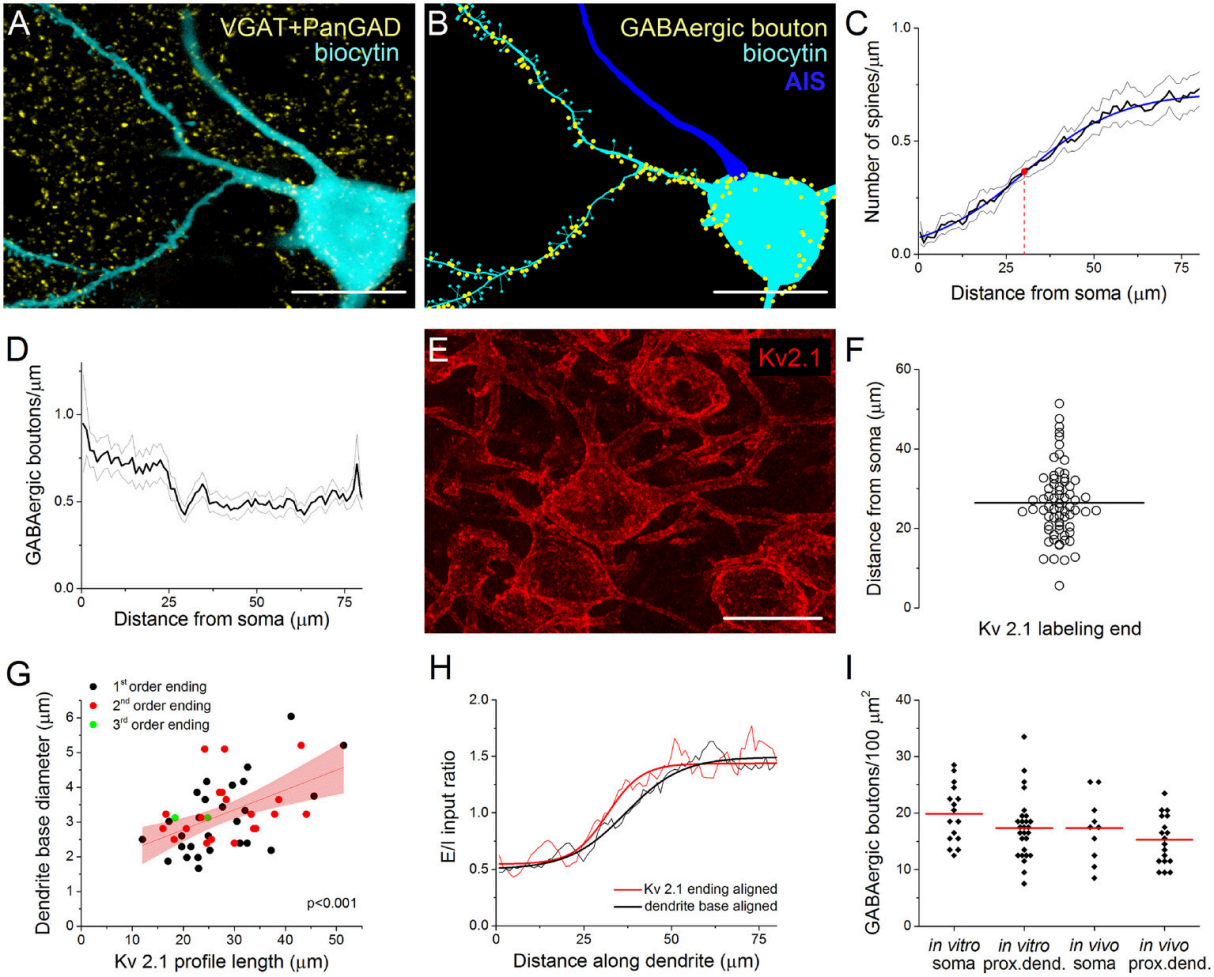
**Immunostaining against Kv2.1 channel protein visualizes the extent of the perisomatic region along the proximal dendrites of principal cells in the BLA, a functional domain receiving predominantly GABAergic inputs. (A)** Maximum z-intensity projection image of a biocytin-filled principal cell (PC) in the BLA shown together with GABAergic boutons visualized with immunostaining against VGAT and PanGAD. **(B)** GABAergic boutons in close apposition with the same PC as in **(A)** are indicated in a Neurolucida reconstruction (AIS, axon initial segment). **(C)** Relationship between the spine number and the distance from the soma. A Boltzmann function (blue line) fitted onto the spine distribution gave an inflection point at 30.5 ± 0.85 μm (red dot). Pooled data obtained from 68 dendrites of 11 PCs. **(D)** The distribution of GABAergic boutons along the dendrites as a function of the distance from the soma (19 dendrites of 4 PCs). **(E)** Kv2.1 immunostaining in the BLA. **(F)** The variance in the length of Kv2.1-immunopositive dendrites is shown. Horizontal line indicates the mean of 26.43 μm (*n* = 68). **(G)** The length of the Kv2.1-labeling correlated with the diameter of biocytin-filled dendrites at their somatic origin, but it was independent from the dendritic branching pattern (i.e., the order of the dendrites). Red area indicates 95% confidence interval of the linear fit. **(H)** Excitatory and inhibitory (E/I) ratios on PCs calculated from data in **(C, D)** are shown aligned by the dendrite base or the end of Kv2.1-immunostained profiles. Note the steeper change in E/I ratio when aligned to the end of Kv2.1-immunolabeled dendrites (see text for details). **(I)** The density of GABAergic boutons on the PC somata and their Kv2.1-immunostained dendrites is similar when determined either *in vitro* and *in vivo*. (red lines: mean of the distributions). Scale bars, 20 μm in **(A, B, E)**.

As in most, if not all cortical structures (Somogyi et al., 1998; Megías et al., 2001), the perisomatic region of PCs in the BLA is exclusively innervated by GABAergic synapses, while glutamatergic synaptic inputs arrive predominantly on their spines or thin dendritic shafts (Carlsen, 1988; McDonald et al., 2002). To indirectly evaluate the glutamatergic inputs, we examined the spine distribution on the dendrites of intracellularly labeled neurons (Figures 1A,B). Using high resolution 3D confocal images, the results of 11 reconstructed PCs showed that the spine number along the dendrites increased gradually (*n* = 68), reaching a maximum density 50–70 μm from the soma. Fitting a Boltzmann sigmoid function onto the spine distribution gave an inflection point (center of the curve) at 30.5 ± 0.85 μm, indicating the distance from the soma where the steepest rise of spine density along the dendrites changes into a modest increase (Figure 1C). This characteristic appearance of the spine distribution along the proximal dendrites of PCs depended on distance rather than a possible dendritic branching pattern, as the spine density along the 1st and 2nd order dendrites changed similarly (data not shown).

#### GABAergic bouton distribution along the proximal dendrites of PCs

To investigate the distribution of GABAergic inputs along the proximal dendrites of biocytin-filled PCs, we visualized the boutons expressing the vesicular GABA transporter VGAT and/or both isoforms of the GABA synthetizing enzyme GAD65/67 (PanGAD) on the membrane surface using immunostaining (Figure 1A). The analysis revealed a decrease in the number of GABAergic varicosities counted to a distance of ~30 μm from the soma, from where the density of immunolabeled boutons showed no further change along the dendrites (*n* = 19 dendrites from 4 PCs, Figures 1B,D).

#### Kv2.1 staining visualizes the portion of proximal dendrites belonging to the perisomatic region of PCs

Earlier work showed that immunostaining against the voltage-gated K^+^ channel subunit Kv2.1 (or KCNB1) labels the perisomatic membrane of neurons (Lim et al., 2000). This raises the possibility that Kv2.1-immunostained segments of the proximal dendrites may actually correspond to those membrane surfaces that belong to the perisomatic region. Indeed, the average length of Kv2.1-immunostained parts of the dendrites (26.43 ± 8.96 μm, *n* = 68, Figure 1E) showed a good correspondence with the inflection point determined by spine distribution (Figure 1C) or by the dendritic length from where the density of GABAergic inputs reached steady state, i.e., no further decrease along the dendrites could be observed (Figure 1D). Moreover, there was a wide variability in the length of the individual Kv2.1-labeled dendritic segments (ranging from 5.6 to 51.4 μm, Figure 1F). Importantly, there was a tight correlation between the length of the Kv2.1-immunostained dendritic segments and the diameter of the dendrites at their somatic origin, which relationship was independent of dendritic order (Figure 1G). These observations prompted us to examine the length of individual Kv2.1-immunostained profiles on those biocytin-filled dendrites where the distribution of the spines and VGAT/PanGAD-immunopositive boutons had been evaluated. Using this approach, we could calculate the ratio of excitatory/inhibitory (E/I) inputs for each individual proximal dendrite by dividing the number of spines with the number of GABAergic boutons along the given dendrite. We hypothesized if there is no relationship between the extent of the Kv2.1 immunoreactivity along the dendrites and the functional boundary of the perisomatic region, then the increase in the E/I input ratio along individual dendrites starting from either the soma or from the distal-most extent of Kv2.1 labeling should have a similar slope. Alternatively, if the extent of the Kv2.1-immunostained dendritic segments corresponds to the border of the perisomatic region, then the increase in the E/I ratio along individual dendrites starting from the distal-most extent the Kv2.1 labeling should have a steeper slope compared to a plot obtained for the E/I ratio along dendrites starting from the base of the dendrites. As shown in Figure 1H, there was a steeper change in E/I input ratio after aligning the data obtained for individual dendrites to the end of the Kv2.1-stained profiles in comparison to that determined with aligning to the base of the dendrites (max. derivative peak: 0.0454 vs. 0.0310 Δ E/I/Δ μm, E/I ratio distributions were significantly different, *p* < 0.001, K-S test; Figure 1H). These results together suggest that the extent of the functional border of the perisomatic region along the individual dendrites of amygdalar PCs shows high variability, and importantly, the perisomatic region of PCs can be visualized by immunostaining against Kv2.1.

Therefore, in the following parts of the study, Kv2.1-immunostained dendrites will be considered as a part of the perisomatic region in addition to the soma and AIS.

### Quantification of GABAergic inputs onto the soma and proximal dendrites of amygdalar principal cells

#### GABAergic boutons opposing soma and proximal dendrites of PCs

We have previously described the number and the spatial organization of inhibitory inputs onto the AIS of PCs in the BLA (Veres et al., 2014). Here, we aimed to reveal the density of GABAergic inputs received by the soma and proximal dendrites of PCs to clarify the quantity of inhibitory inputs contacting the perisomatic region. To this end, we determined the number of VGAT/PanGAD-immunoreactive boutons closely opposed to the soma and Kv2.1-immunostained dendrites of biocytin-filled PCs. To validate the data obtained in *in vitro* slices, we similarly determined the number of GABAergic inputs onto the perisomatic regions of PCs in perfused tissue. The results showed that ~17–20 boutons/100 μm^2^ could be found on the surface of the soma both *in vitro* and *in vivo* (19.87 ± 5.18/100 μm^2^ and 17.32 ± 5.81/100 μm^2^, *n* = 16 and 10, respectively), while the density of GABAergic inputs was slightly less on the proximal dendrites (*in vitro*: 17.34 ± 5.56/100 μm^2^ and *in vivo*: 15.25 ± 4.36/100 μm^2^, *n* = 27 and 18, respectively, Figure 1I). As the total soma surface of Kv2.1-labeled PCs was found to be 881.4 ± 83.4 μm^2^ on average (*n* = 10, range: 735.1–1030.7 μm^2^), while the surface of the proximal dendrites was 439.8 ± 128.2 μm^2^ (49 dendrites of 8 PCs), we calculated that ~158 and 71 GABAergic boutons form close contacts with the soma and proximal, Kv2.1-immunostained dendritic segments of amygdalar PCs, respectively.

#### No evidence for glutamatergic boutons opposing soma and proximal dendrites of PCs

To confirm the earlier observation that the perisomatic region is not targeted by glutamatergic inputs, we performed immunostaining against type 1 and type 2 vesicular glutamate transporters (VGLUT1 and VGLUT2) together with bassoon staining on biocytin-filled PCs. The former two markers are known to visualize the excitatory inputs (Takamori et al., 2000, 2001), while the presence of bassoon helps to identify the release sites in the boutons (Papp et al., 2013). The analysis of this immunostained material revealed no VGLUT1- or VGLUT2-immunoreactive bouton, which bassoon-immunolabeled portion opposed toward the soma of biocytin-filled PCs (*n* = 7), while such terminals could be readily observed to contact PV-expressing cell bodies (*n* = 3, unpublished observations). This latter observation is in accord with earlier studies describing that these interneurons receive excitatory synapses on their cell bodies (Gulyás et al., 1999; McDonald et al., 2005), and verify the previous results that the perisomatic region of PCs in the BLA receive only an insignificant number of excitatory inputs.

### The vast majority of GABAergic inputs onto the soma and proximal dendrites of principal cells originates from two distinct types of GABAergic interneurons

#### Quantification of PV- and CB_1_-expressing boutons contacting soma and proximal dendrites of PCs

**Figure 2:**
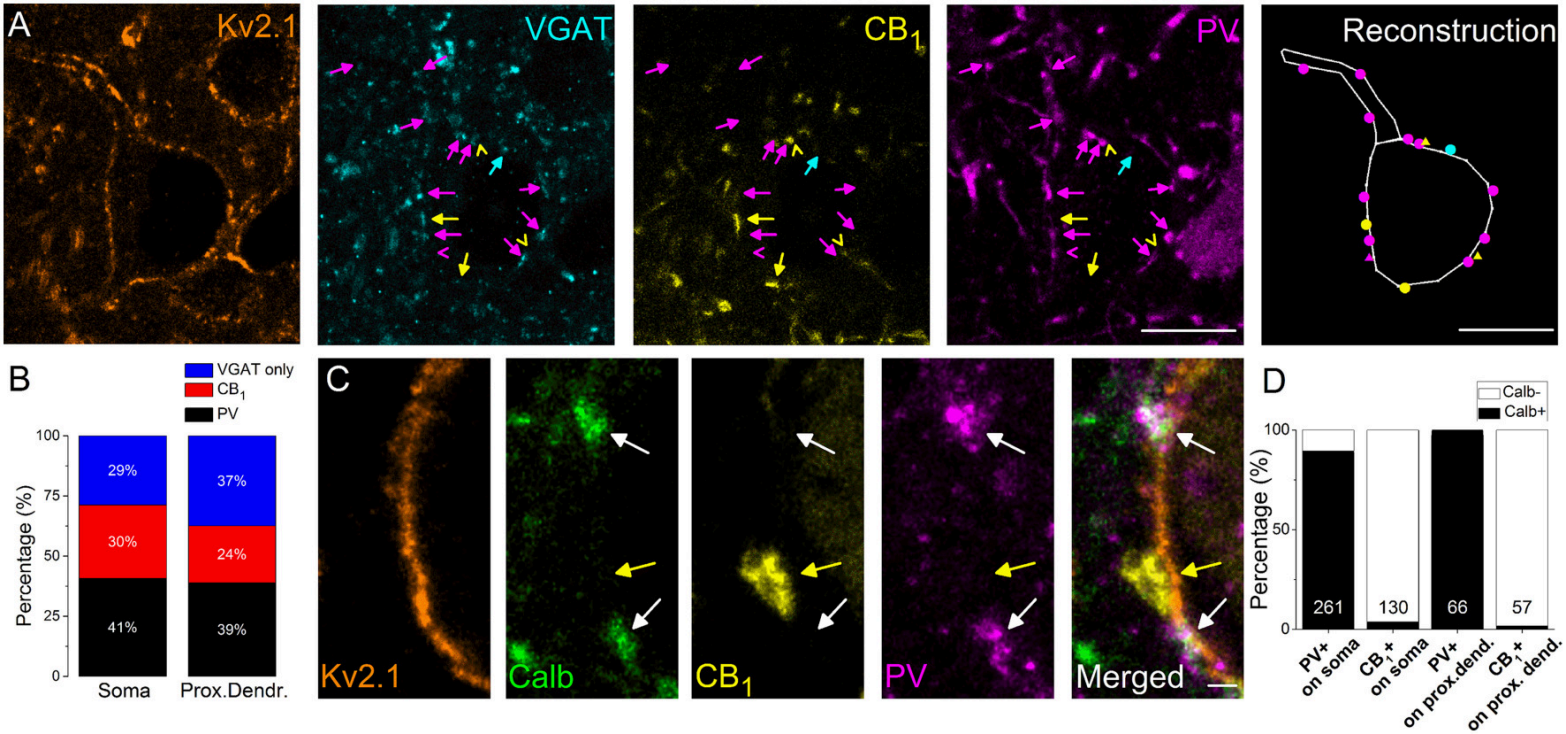
**The vast majority of perisomatic GABAergic inputs onto the principal cells originates from boutons co-expressing parvalbumin (PV) and calbindin (Calb) or CB_**1**_ cannabinoid receptors. (A)** Multicolor single plane confocal images taken from immunostaining against Kv2.1 (to delineate the perisomatic region of PCs), VGAT, CB_1_, and PV. Far right: the reconstruction of a Kv2.1-immunostained neuron receiving GABAergic inputs. Arrows and dots in magenta point to VGAT/PV-immunostained boutons, an arrowhead, and a triangle in magenta mark an only PV-containing bouton, arrows, and dots in yellow indicate VGAT/CB_1_-immunopositive varicosities, arrowheads, and triangles in yellow mark only CB_1_-immunoreactive boutons and an arrow and a dot in blue show an only VGAT-immunolabeled bouton forming close appositions with a Kv2.1-immunostained neuron. **(B)** Ratio of boutons expressing PV, CB_1_, and VGAT only on the Kv2.1-immunostained soma and proximal dendrites of neurons. **(C)** Multicolor single plane confocal images taken from immunostaining against Kv2.1, Calb, CB_1_, and PV. White arrows point to boutons co-expressing Calb and PV forming close appositions with a Kv2.1 immunostained soma membrane segment, while a yellow arrow indicates a CB_1_-immunoreactive varicosity lacking Calb immunolabeling. **(D)** Ratio of PV- or CB_1_-expressing boutons immunoreactive for Calb on soma or proximal dendrites. Numbers of boutons evaluated are indicated for each column. Scale bars, 10 μm in **(A)**, 1 μm in **(C)**.

In the next set of experiments, we intended to reveal the interneuron types contributing to the GABAergic inputs received by the soma and proximal dendrites of amygdalar PCs. As prior studies elucidated, inhibitory axon terminals expressing either PV or CB_1_ formed synaptic contacts with the perisomatic membrane of PCs (Smith et al., 1998; Katona et al., 2001; McDonald et al., 2005). Therefore, using multicolor immunofluorescent stainings, we examined the ratio of PV- and CB_1_-immunoreactive boutons among VGAT-immunolabeled varicosities in close apposition to the perisomatic membranes of PCs (Figure 2A). We found that (1) 67% of VGAT-immunostained boutons both on the soma and the Kv2.1-immunoreactive dendrites were immunopositive for PV or CB_1_ (*n* = 498 boutons examined on 10 soma and 234 boutons on 18 dendrites, Figure 2B) and (2) more PV- than CB_1_-immunoreactive boutons contacting the perisomatic region could be observed (*p* = 0.007, M-W test), Figure 2B).

#### Calb content distinguishes PV- and CB_1_-expressing boutons contacting soma and proximal dendrites of PCs

In addition, we examined the Calb content of PV- or CB_1_-immunostained boutons because previous studies in the amygdala have shown that Calb-immunoreactive axon terminals could also form symmetrical synapses with the perisomatic membranes of PCs (Muller et al., 2003). Using multicolor immunofluorescent stainings we clarified that Calb immunoreactivity was present predominantly in PV-immunostained varicosities (91.7% of all PV-immunopositive boutons also contained Calb, *n* = 300∕327 boutons), but was negligible (2.7%, *n* = 5∕187 boutons) in CB_1_-expressing boutons opposing the membrane surface of the perisomatic region (Figures 2C,D). These data show that most (67%) of the GABAergic inputs onto the soma and proximal dendrites of PCs originates from two interneuron populations, i.e., from two distinct types of basket cells (BCs), which differ in their Calb content.

### Interneurons innervating the perisomatic region of amygdalar principal cells are neurochemically distinct

#### Targeting the three perisomatic region-innervating interneurons in slices

**Figure 3:**
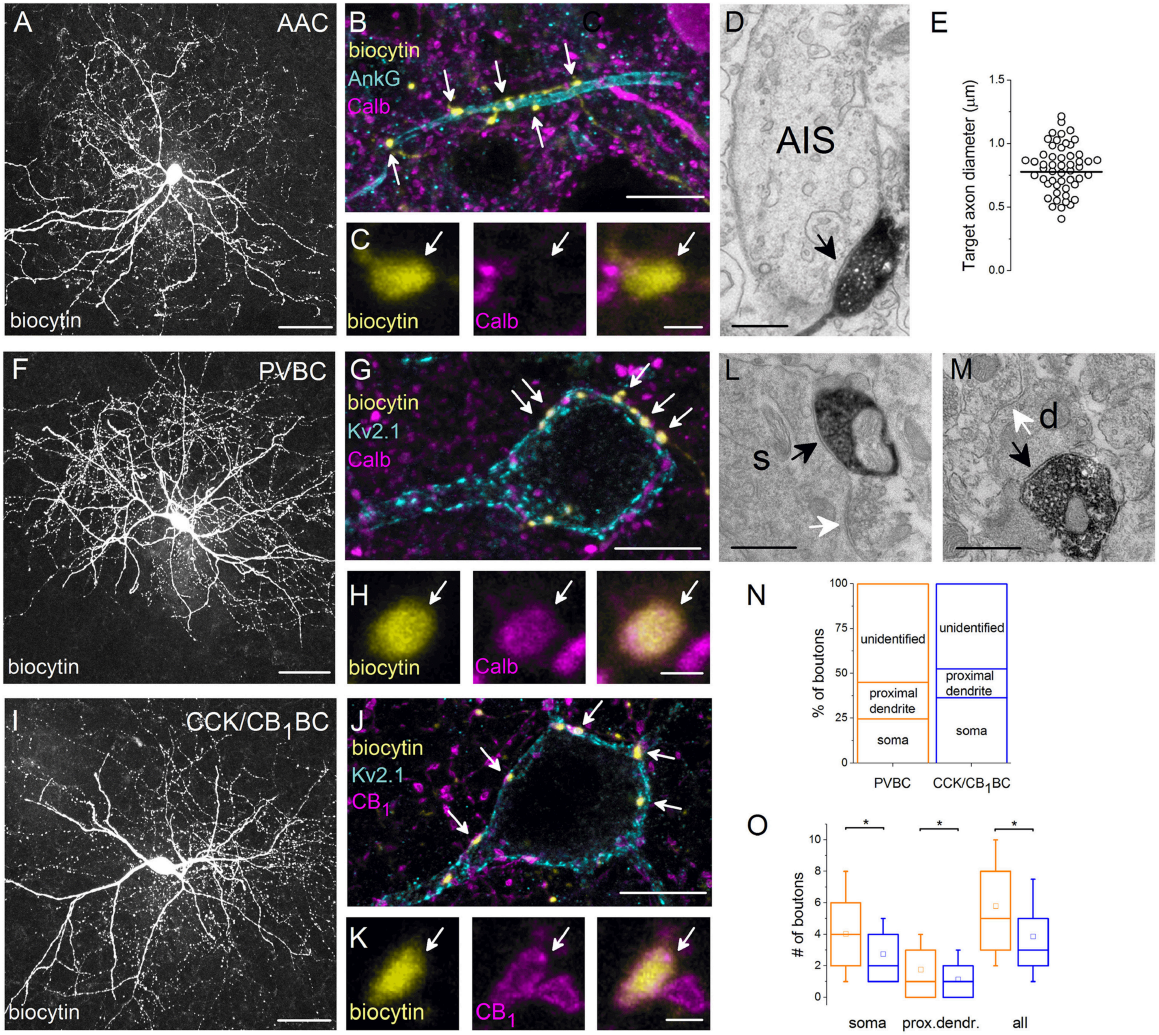
**Neurochemical content and postsynaptic targets of interneurons innervating the perisomatic region of principal cells in the BLA**. Maximum z intensity projection images taken of an *in vitro* biocytin-filled AAC **(A)**, PVBC **(F)**, or CCK/CB_1_BC **(I)**. **(B)** Varicosities of the AAC in **(A)** contact with an AIS visualized by ankyrin G staining and lack Calb immunoreactivity **(C)**. **(D)** A biocytin-filled bouton of an AAC forms a symmetrical synapse on an AIS. **(E)** Distribution of target axon diameters obtained by random sampling (55 boutons from 7 AACs). Black lines represent the mean value. **(G)** The biocytin-containing boutons of the same cell as in **(F)** form close contacts with the Kv2.1-labeled perisomatic region of a PC and express Calb **(H)**. **(J)** The boutons of the interneuron in **(I)** form close appositions with the Kv2.1-immunostained membranes of a PC and express CB_1_ **(K)**. **(L, M)** Electron micrographs show biocytin-labeled axon terminals of a PVBC forming symmetrical synapses on a soma (s) or a small caliber dendrite (d; black arrows). The same postsynaptic elements also received symmetrical synapses from unlabeled axon endings (white arrows). **(N)** Ratio of boutons of PVBCs (*n* = 12) and CCK/CB_1_BCs (*n* = 12) forming close contacts with Kv2.1-immunostained somata, proximal dendrites, or which did not appose any Kv2.1-immunostained profiles (unidentified). Note, that the high ratio of boutons contacting the perisomatic region of PCs defines the cells as BCs. **(O)** A larger number of boutons from PVBCs (orange) than from CCK/CB_1_BCs (blue) contact the perisomatic region of individual PCs. Asterisks mark significant differences (for values see the text). The mean (small open square), the median (midline of the box), the interquartile range (box), and the 5/95% values (ends of whisker bars) are plotted. AAC, axo-axonic cell; PVBC, parvalbumin-containing basket cell; CCK/CB_1_BC, cholecystokinin and CB_1_ cannabinoid receptor-expressing basket cell. Scale bars, 50 μm in **(A,F,I)**, 10 μm in **(B,G,J)**; 1 μm in **(C,H,K)**; 0.5 μm in **(D,L,M)**.

Up to this point, we have focused on the GABAergic innervations of the soma and the proximal dendrites of PCs. However, the AIS is also considered as a part of the perisomatic region. In our recent work (Veres et al., 2014), we established that AACs expressing PV gave rise to the vast majority of the GABAergic inputs onto the AIS. Here, we aimed to compare and contrast the morphological and neurochemical properties of all major interneuron types innervating the perisomatic region, i.e., AACs and two types of BC. To achieve this aim, PV- and CB_1_-expressing interneurons, which were shown to give rise to the majority of GABAergic inputs onto the perisomatic membrane surface of PCs (Figure 2), were intracellularly-labeled in slices prepared from PV-eGFP and CCK-DsRed mice, respectively. In PV-eGFP mice, although both AACs and PVBCs were sampled, these cell types could be separated *post-hoc* at the light microscopic level using double immunofluorescent staining against biocytin and ankyrin G (PVBC, *n* = 50; AAC, *n* = 32). The validity of this method was confirmed using electron microscopy (Figures 3A,B,D,F,L,M). In case of interneurons sampled in CCK-DsRed mouse slices, expression of CB_1_ on the axon terminals of each recorded cell was verified using immunostaining (*n* = 32, Figures 3I–K) to unequivocally identify this interneuron type. After morphological identification, a total of 114 identified interneurons were included in the study.

In all cases, the interneurons labeled with biocytin had spine-free or rarely spiny dendrites and dense local, often ramified axon collaterals bearing large boutons (Figures 3A,F,I). Both the dendritic and axonal branches of intracellularly-filled interneurons were mainly restricted to the amygdala, rarely penetrating into the capsules surrounding this cortical area.

#### Different expression of calb in PVBCs and AACs

Using multicolor immunofluorescent stainings, we first examined the Calb content of anatomically identified interneurons. We found that Calb content clearly distinguished PV-expressing interneurons: PVBCs expressed Calb both in their boutons and cell bodies (*n* = 25 and 16 cells examined, respectively, Figure 3H), while the soma of all but one AAC were found to be immunonegative (*n* = 14), and no immunolabeling for Calb in the axon terminals of examined AACs could be detected (*n* = 18 cell investigated, Figure 3C). The CB_1_-immunostained varicosities of biocytin-filled interneurons recorded in CCK-DsRed mice, i.e., CCK/CB_1_BCs were also found to be Calb immunonegative (*n* = 10, data not shown). These results confirmed and extended our data obtained in perfused tissue (Figure 2), showing that among interneurons targeting the perisomatic region, only PVBCs express Calb.

### Output characteristics of the three perisomatic region-targeting interneuron types in the BLA

#### Postsynaptic targets of AACs

In the following part of the study, we aimed at getting a deeper insight into the target distribution of individual interneurons innervating the perisomatic region of PCs. First, we re-analyzed the postsynaptic target distribution of AACs using electron microscopy. In line with previous studies (Bienvenu et al., 2012; Veres et al., 2014) we found that axon terminals of AACs (*n* = 7) formed symmetrical synapses predominantly on the AIS (Figure 3D), but not with somata or dendrites. By measuring the smallest diameter of the postsynaptic target profiles, we found that 74% of randomly sampled axon varicosities (41 of 55) targeted axon segments having a diameter between 0.5 and 1 μm (Figure 3E). To compare these data with our previous results (Veres et al., 2014), we determined the smallest diameter of AISs where the axon terminals of AACs (*n* = 3) formed synaptic contacts with identified PCs in paired recordings (*n* = 25). We observed that (1) there was no significant difference in the diameter of targets obtained in paired recordings (Veres et al., 2014) or in those that were randomly sampled (present study; random sampled: 0.777 ± 0.218 μm; in pairs: 0.872 ± 0.196 μm, *p* = 0.11, M-W test), (2) the most proximal part of the axon (<10 μm) was rarely contacted by AACs, as only one bouton was found to form synapses on a profile having a larger diameter than 1.25 μm and (3) AACs might also target distal axons, as some boutons (*n* = 5; 9%) contacted a postsynaptic profile having a smaller diameter than 0.5 μm (the postsynaptic targets lacked spines and had a dense intracellular matrix often containing vesicles, characteristic for axons). These results confirmed our previous observations that AACs do not contact uniformly along the initial part of axons, but selectively target a particular portion, the diameter of which ranges from 0.5 to 1 μm and may also form synapses on distal axons (i.e., beyond the extent of the AIS).

#### Target distribution of BCs

Next, we examined the potential targets of both BC types using light microscopy. If a biocytin-containing bouton formed a close apposition with a Kv2.1-immunostained soma or dendritic segment (Figures 3G,J), then this bouton was considered to target the perisomatic region. If no Kv2.1-immunostained profile could be seen in the close vicinity of a biocytin-filled bouton, then a non-perisomatic/unidentified target was marked. To reveal whether those boutons contacting non-Kv2.1-immunostained profiles could form synaptic contacts presumably on distal dendrites, electron microscopic examination of randomly sampled biocytin-filled varicosities was carried out. We verified that dendrites in addition to somata and proximal dendrites were among the targets of biocytin-filled boutons (PVBC: 23/57 synapses on dendrites (40%), CCK/CB_1_BC:32/54 synapses on dendrites (59.2%), *n* = 6 and 5 cells, respectively, Figures 3L,M). These data indicate that boutons avoiding Kv2.1-immunostained segments could target more distal dendrites of PCs. Having this in mind, we estimated the distribution of potential targets for BCs. Using this light microscopic approach, we found that the boutons of PVBCs (2529 boutons, *n* = 12 cells) and CCK/CB_1_BCs (2642 boutons, *n* = 12 cells) innervated the perisomatic region of PCs with similar probability (*p* = 0.25, *t*-test, Figure 3N), although the ratio of those varicosities that targeted only the somata was significantly larger for CCK/CB_1_BCs than for PVBCs (*p* = 0.02, *t*-test).

#### Convergence of BCs onto single PCs

In addition to the target distribution, this approach allowed us to estimate the convergence of GABAergic inputs onto single PCs from the two types of BC. Therefore, we counted those boutons originating from individually labeled interneurons that formed close appositions with the soma and proximal dendrites of single Kv2.1-immunolabeled PCs. We found, that individual PVBCs contacted single PC somata with 31% more boutons than CCK/CB_1_BCs (PVBC: 4.04 ± 2.90 boutons/individual soma, 481 boutons on 119 PC cell bodies from 8 interneurons; CCK/CB_1_BC: 2.76 ± 1.96 boutons/individual soma, 357 boutons on 129 PC somata from 8 interneurons, *p* < 0.0001, M-W test, Figure 3O). In addition, the difference in the number of boutons contacting Kv2.1-immunoreactive dendritic profiles was lower, but statistically significant (PVBC: 1.76 ± 1.80 boutons/proximal dendrite of individual cells, 210 boutons on 119 PCs from 8 interneurons; CCK/CB_1_BC: 1.14 ± 1.29 boutons/proximal dendrite of individual cells, 148 boutons on 129 PCs from 8 interneurons, *p* = 0.0005, M-W test, Figure 3O). Merging these data showed that the perisomatic region of single PCs received 33% more boutons from individual PVBCs than from individual CCK/CB_1_BCs (PVBC: 5.80 ± 3.33, CCK/CB_1_BC: 3.91 ± 2.66, *p* < 0.0001, M-W test), Figure 3O). Taking into account that single PCs receive on average 93 PV- and 64 CB_1_-immunopositive varicosities on their perisomatic region (Figure 2B), these findings indicate that approximately the same number of PVBCs (15–16) and CCK/CB_1_BCs (16–17) converge onto individual PCs in the BLA (**Figure 6**).

#### PVBCs target the proximal portion of PC axons

**Figure 4:**
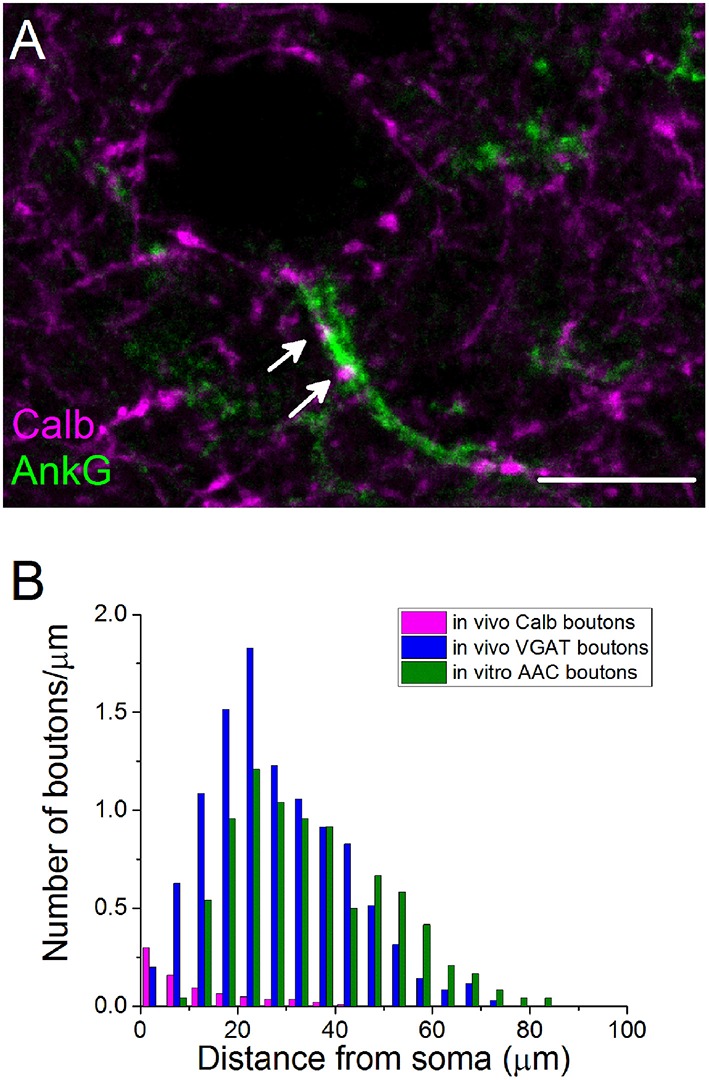
**The initial part of principal cell axons is parceled out by PVBCs and AACs. (A)** A confocal image taken from immunostaining for Calb and ankyrin G. Arrows indicate Calb-containing boutons on an AIS. **(B)** The number of Calb-immunoreactive boutons shows a steep decrease from the soma toward the end of the anykrin G-immunolabeled AISs. For comparison, the spatial distribution of total number of GABAergic varicosities and boutons of AACs taken from Veres et al. (2014) are shown in the same plot. Note, that Calb-positive axon terminals of PVBCs and boutons of AACs parcel out the AISs. Scale bar, 10 μm.

Previous studies in the rat hippocampus raised the possibility that BCs could occasionally target the AISs of PCs (Halasy et al., 1996), but neither the ratio of BC boutons among GABAergic inputs contacting AISs nor their spatial occurrence along the proximal part of the axons are known. Therefore, we intended to estimate the number and the distribution of BC boutons along the initial part of the axons by exploiting the finding that the Calb content of varicosities originating from PVBCs distinguishes them from those that belong to AACs (Figures 3C,H). By examining the distribution of Calb-immunoreactive boutons along the ankyrin G-stained profiles in perfused tissue samples, we found that 60% of those PVBC boutons that formed close appositions with axons contacted the proximal 10 μm-long portion of the axon (*n* = 107 Calb-containing boutons, Figure 4A). Importantly, the number of Calb-immunoreactive boutons decreased sharply toward the end of the AISs (Figure 4B). When we plotted these data together with those published in our recent study, where the total number of GABAergic inputs along the initial part of axons was compared to those originated from identified AACs (Veres et al., 2014), it was clearly visible that the proximal part of PC axons was predominantly innervated by PVBCs. Notably, as the amount of Calb-immunoreactive boutons along the AISs decreased, the number of AAC inputs increased, showing a complementary innervation of axon origin by these two cell types (Figure 4B). In addition to the spatial distribution of PVBC boutons along the proximal part of the axons, we could also estimate the ratio of these varicosities in comparison with AAC inputs. As we found earlier (Veres et al., 2014), ~50–60 GABAergic varicosities contact with the axon origin of single PCs: therefore 6–7 % of the total GABAergic inputs onto this membrane compartment could arise from PVBCs (3.8 ± 1.9 Calb-positive bouton/AIS, *n* = 28 AISs). In separate double-immunostained material we found no close appositions between CB_1_-expressing boutons and ankyrin G-stained profiles (*n* = 10 AISs, data not shown), indicating that the output of CCK/CB_1_BCs contributes negligibly to the GABAergic innervation of AISs. These results elucidated that, in line with our electron microscopic analysis (Figure 3E), the very proximal part of PC axons (the first 10 μm), where the immunostaining against voltage-gated Na^+^ channel type 1.6 is low (Veres et al., 2014), is avoided by AACs but mainly innervated by PVBCs, indicating that this proportion of the axon might be equivalent to the axon hillock, and considered as a soma-equivalent membrane surface (Kole and Stuart, 2012).

### Basket cells comprise 75% of PV-expressing interneurons in the BLA

In addition to the varicosities, the Calb content in the soma of PV-containing cells discriminates BCs from AACs, allowing us to estimate the ratio of these two interneuron types among PV-expressing interneurons in the BLA at the population level. By evaluating the proportion of Calb-immunoreactive and immunonegative somata among PV-expressing interneurons in the BLA in perfused tissue samples, we found that 26.1% of PV-immunoreactive cell bodies lacked Calb content (140 out of 537, *n* = 3 animals). These data suggest that three quarters of PV-expressing interneurons in the BLA are BCs, while every fourth interneuron immunostained for PV is likely to be an AAC.

### Density of the two basket cell types in the BLA is similar

Our convergence data showed that the same number of BCs expressing PV or CCK/CB_1_ innervate individual PCs in the BLA, suggesting that a similar number of BCs from both types should be present in this brain area. To verify this assumption, we calculated the density of PVBCs and CCK/CB_1_BCs in perfused tissue samples. We found that PV- and CCK-containing interneurons had a density of 1.81 × 10^3^ ± 0.92 × 10^3^ /mm^3^ and 2.03 × 10^3^ ± 0.74 × 10^3^ /mm^3^, respectively (*n* = 5–10 section from 2 mice). If we assume that 75% of all PV-expressing interneurons are BCs, then the density of PVBCs in the BLA is 1.36 × 10^3^ ± 0.69 × 10^3^ /mm^3^. Previous studies described that 30-40% of CCK-expressing interneurons could be Calb immunoreactive in the rat amygdala (Mascagni and McDonald, 2003). Because no Calb immunolabeling in the boutons of CCK/CB_1_BCs has been detected (Figure 2), in line with data obtained in the hippocampus (Cope et al., 2002; Klausberger et al., 2005), we suppose that CCK-containing interneurons lacking Calb should be BCs. To uncover the colocalization ratio of Calb in CCK-expressing interneurons in the mouse BLA, we performed double immunofluorescent staining. Our analysis revealed that 30% of the somata of all CCK-containing interneurons expressed Calb (19 from 63 cells evaluated). Thus, 70% (or less) of CCK-expressing interneurons should be BCs. Using this ratio to estimate the density of CCK/CB_1_BCs in the BLA, the calculation provides a density of 1.31 × 10^3^ ± 0.86 × 10^3^ /mm^3^ for these interneurons. Thus, our investigations showed that a similar number of the two types of BC is present in the BLA. These results obtained by an independent approach strongly support our conclusion that the same number of PVBCs and CCK/CB_1_BCs should contact single PCs in the BLA.

### Comparison of dendritic and axonal arborization of perisomatic region-targeting interneurons

**Figure 5:**
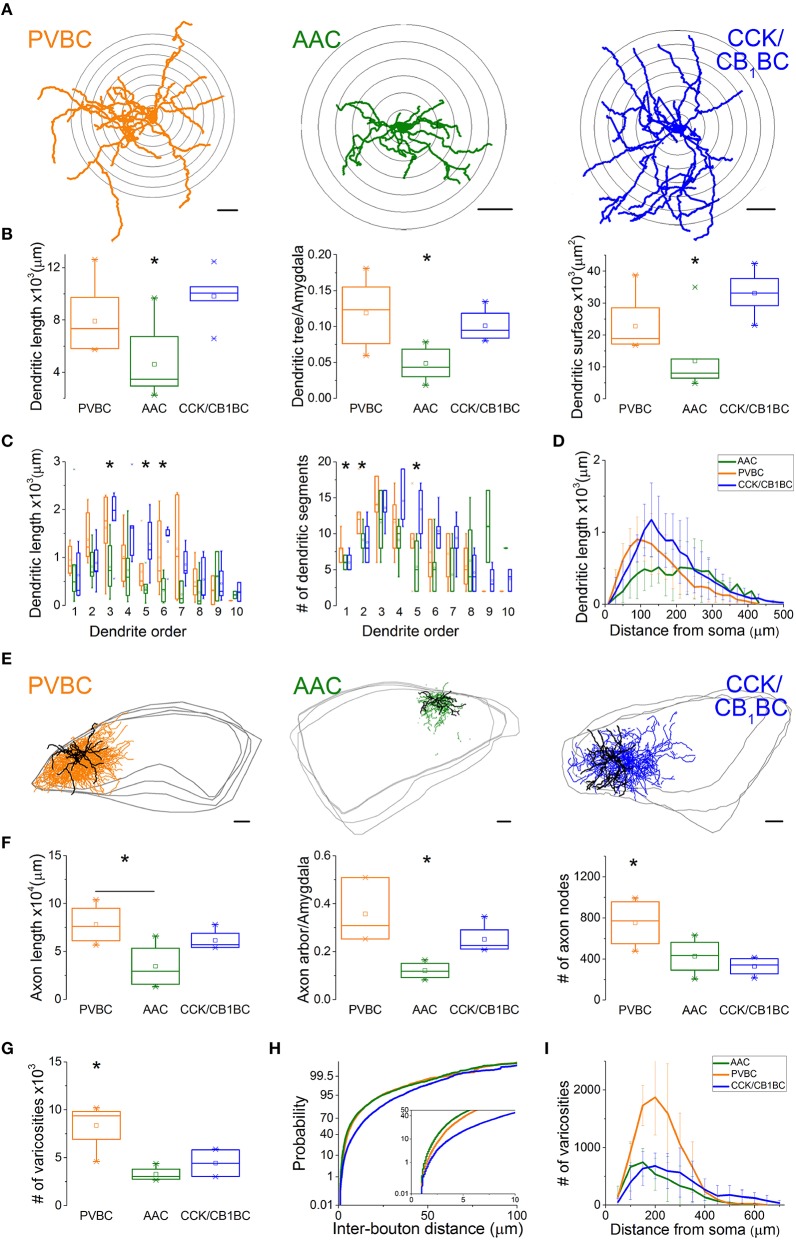
**Dendritic and axonal arborization of the three perisomatic region-targeting interneurons are distinct. (A)** Neurolucida reconstructions of the dendritic arbor of three example cells labeled in slice preparations. Concentric circles drawn on the reconstructions illustrate the radii used for Sholl Analysis. **(B)** Box chart comparison of total dendritic length, the ratio of the extent of the dendritic tree and the amygdala within the slices, and total dendritic surface. The mean (small open square), the median (midline of the box), the interquartile range (box), and the 5/95% values (ends of whisker bars) are plotted. **(C)** Comparison of the dendritic length and the number of dendritic segments as a function of dendritic order. **(D)** Dendritic length as a function of distance from the soma. **(E)** Neurolucida reconstructions of the biocytin-filled processes (dendrites in black, axon in color) of the same interneurons as in **(A)**. The borders of the amygdala are shown in gray. **(F)** Box chart comparison of total axon length, the ratio of the extent of the axon arbor and the amygdala within the slices, and the number of axon nodes. Box and whiskers as panel **(B)**. **(G)** Comparison of the number of varicosities on axon collaterals. Box and whiskers as panel **(B). (H)** Cumulative probability distributions of the inter-bouton distances for the three cell types. The initial 10 μm of the distribution is enlarged in the inset. **(I)** Number of varicosities as a function of soma distance. For more details, see Table 2. All scale bars are 100 μm. ^*^indicates significant differences.

After revealing the postsynaptic targets and the neurochemical content of recorded interneurons, we explored their dendritic and axonal arborization features to compare the structural basis of the input and output properties of perisomatic region-targeting interneurons in the BLA. Using Neurolucida software, we reconstructed the dendritic tree and axonal arbor and counted the varicosities along the axon collaterals (Figure 5). The comparison of the dendritic trees showed that AACs had the shortest dendrites, which occupied about 5% of the amygdala in slices, while both BC types emitted longer dendritic branches covering about 10% of the amygdala (Figure 5B, Table 2). In parallel, the surface of the dendrites of AACs was also significantly smaller (Figure 5B, Table 2). In the structure of the dendritic trees we also found some differences (Figure 5C). First, PVBCs emitted more primary and secondary dendrites than the other two cell types. Second, as shown in Figure 5C, CCK/CB_1_BCs had more higher-order dendritic segments, which together were longer than those observed for the other two cell types, resulting in a longer dendritic length further away from the soma than that seen for PVBCs (Figure 5D). We also observed differences in the axon characteristics of the examined interneurons. As in the case of the dendrites, AACs had the shortest axon, which covered about 10% of the amygdala within the *in vitro* slice (Figure 5F, Table 2). In contrast, the axon arbor of BCs occupied around 30% of the amygdala within the slice. PVBCs had the most ramified axon arbor, indicated by the number of nodes (Figure 5F, Table 2). Although the total axon length was not different between BCs, the number of varicosities was significantly higher in the case of PVBCs (Figure 5G, Table 2), in agreement with the finding that the mean inter-bouton distance for CCK/CB_1_BCs was significantly larger than for the other two cell types (Figure 5H, Table 2). Although the number of boutons of the two BC types had similar maximum values around 200 μm from their soma, the density of CCK/CB_1_BC varicosities showed a more even distribution with distance (Figure 5I). The varicosities of AACs were more numerous closer to their soma in comparison with BCs (Figure 5I). These observations collectively suggest that, based on detailed morphological analysis, the input-output properties of the three perisomatic region-targeting interneurons are differently structured.

### Interneuron output features in the BLA

#### Divergence of the three interneuron types

**Figure 6:**
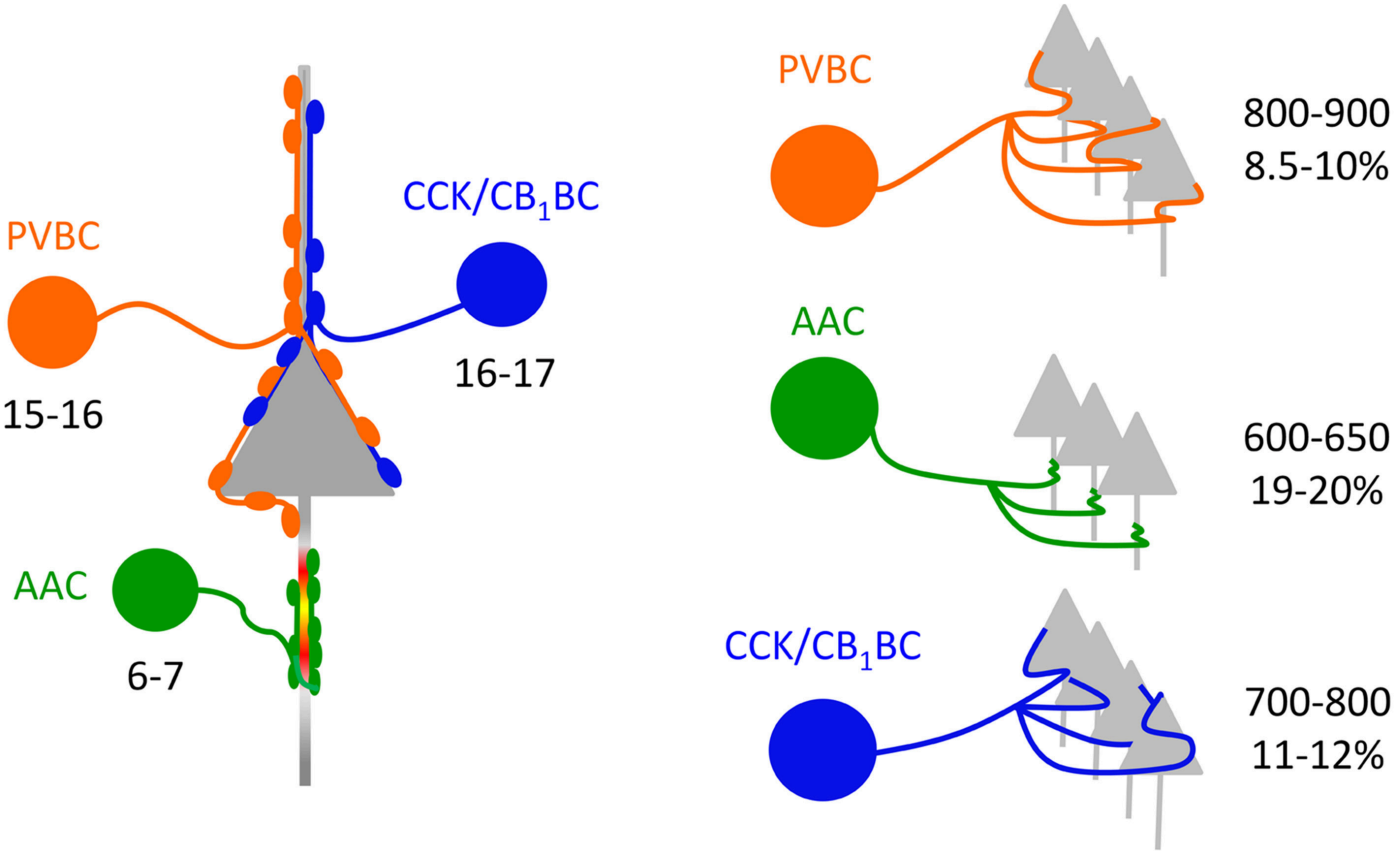
**Convergence and divergence in the microcircuits composed of perisomatic region-targeting interneurons and principal cells in the BLA**. Left, Similar numbers of PVBCs and CCK/CB_1_BCs converge on a PC in the BLA. In contrast, fewer AACs innervate the axon initial segment of single PCs (data from Veres et al., 2014). Right, The quantity of PCs targeted by singe BCs is comparable, while individual AACs innervate fewer PCs. The percentage refers to the fraction of PCs that are innervated by single interneurons within their axonal cloud.

Based on the estimate that 60–65% of the total arborization of individual interneurons could be recovered in 300-μm-thick slices (see Materials and Methods), we could approximate the divergence of interneurons in the BLA. Our data showed that a PVBC and a CCK/CB_1_BC innervate the perisomatic region of single PCs via 5.8 and 3.9 contacts, respectively (Figure 3O), and that 45% (PVBCs) and 52% (CCK/CB_1_BCs) of their boutons target the perisomatic region on average (Figure 3N). These data together implicate that a PVBC and a CCK/CB_1_BC innervate single PCs via 12–13 and 7–8 contacts, respectively. Combining these data with the bouton number obtained in single cell reconstructions (Figure 5G, Table 2), a PVBC and a CCK/CB_1_BC may target 1000-1100 and 900-1000 neurons, respectively, within their projection area. If in the BLA of the mouse roughly 20% of the neurons are GABAergic cells (McDonald, 1985; McDonald and Augustine, 1993) and these cell types are targeted by BCs via a similar number of contacts as PCs receive, then a PVBC and a CCK/CB_1_BC may target 800–900 and 700–800 PCs, respectively (Figure 6). As an AAC contacts single PCs via 8.4 synapses on average (Veres et al., 2014), this interneuron type bearing 5000–5500 axon varicosities on its total axon collaterals can innervate 600–650 PCs (Figure 6).

#### Fraction of PCs innervated by single interneurons

To estimate the fraction of PCs that can be innervated by single interneurons within the area occupied by their axons, the number of PCs in this area should be determined. Using stereology, we found that the total number of neurons within the lateral, basolateral, and basomedial parts of the amygdala was ~70,000 (70,076 ± 8053, *n* = 3 mice). We approximated that within a 300-μm-thick amygdalar slice 26,200 PCs can be present. Taking into account the differences observed in the area covered by the axon collaterals of distinct interneuron types (Figure 5F, Table 2) and the number of potential targets per interneuron (see above), we found that a PVBC may target 8.5–10%, an AAC may innervate 19–20%, and CCK/CB_1_BCs may contact with 11–12% of all PCs within its axon cloud (Figure 6). However, it should be noted that the number of the terminals closer to the IN somata is higher than more distally (with a peak around 200 μm, Figure 5I), which implies that the fraction of the innervated PCs is also higher at the close proximity of the INs. The comparable fraction of the total number of PCs innervated by BCs within their axon arbor shows that these interneuron types can have a similar strategy to select the postsynaptic targets within the amygdalar network, while the target selection of AAC may follow distinct rules.

## Discussion

Our main findings are as follows: (1) The soma and proximal dendrites of PCs are innervated primarily by two morphologically and neurochemically distinct BC types. (2) Similar numbers of PVBCs and CCK/CB_1_BCs converge onto single PCs, although individual PVBCs contact the perisomatic region of PCs via more boutons than CCK/CB_1_BCs. (3) The initial part of PC axons is parceled out by PVBCs and AACs, as the majority of GABAergic inputs onto the proximal region (between 0 and 10 μm) originates from PVBCs, while the largest portion of the AIS is innervated by AACs. (4) Significant differences in the extent and the structure of dendritic and axonal arborization of the three perisomatic region-targeting interneurons were found. Combined, these results suggest that the three main perisomatic region-targeting interneuron types are differentially affiliated within the local amygdalar circuitry, and therefore they can fulfill specific functions in network operation during various brain states.

Our data suggest that Kv2.1 immunostaining, at least for PCs in the BLA, can be an appropriate tool to reveal the extent of the proximal dendritic segments that belong to the perisomatic region. Earlier studies obtained in the hippocampus (Bannister and Larkman, 1995; Megías et al., 2001; Papp et al., 2001), in the neocortex (Ballesteros-Yáñez et al., 2006) or recently in the amygdala (Padival et al., 2013; Klenowski et al., 2015; Ryan et al., 2016) uncovered similar increases in spine density along the proximal dendrites of PCs measured from the soma as we observed here. Importantly, Papp et al. (2001) compared the spine density and number of GABAergic inputs along the apical dendrites of CA1 pyramidal cells and found that these two structures showed parallel anti-correlated changes in quantity with distance from the soma, just as we have uncovered in the present study in the BLA (Figures 1C,D). Collectively, these results suggest that the organizational principle of excitatory and inhibitory inputs along the proximal part of the PC dendrites might be similar in different cortical regions.

### Perisomatic innervation of PCs in the BLA

We found that the density of GABAergic boutons on the PC soma surface was comparable to that observed for CA1 pyramidal cells in the mouse hippocampus (Lee et al., 2014; Takács et al., 2015), but was twice as much as reported for rat neocortical pyramidal cells (Wolff and Chronwall, 1982) or even half in other studies obtained in cats (Davis and Sterling, 1979; Fariñas and DeFelipe, 1991) or recently in the rat amygdala (Klenowski et al., 2015). Thus, the species difference might be one of the main reasons, which might account for the discrepancy found in perisomatic innervation by different groups.

Earlier work established that GABAergic boutons expressing PV, CB_1_ or Calb formed symmetrical synapses with the perikarya and proximal dendrites of PCs in amygdala (Smith et al., 1998; Katona et al., 2001; Muller et al., 2003, 2006). We confirmed these results and extended them by showing that PV and Calb were colocalized in the same varicosities contacting the Kv2.1-immunostained profiles, and we found these boutons to originate from PVBCs. In addition, CB_1_-expressing boutons forming synapses with the perisomatic region derived from CCK/CB_1_BCs, and were found to lack Calb immunolabeling. In contrast to the hippocampus, where almost exclusively BCs expressing PV or CB_1_ innervate the perikarya of pyramidal cells (Takács et al., 2015), we found that ~30% of GABAergic boutons contacting the Kv2.1- immunostained profiles was immunonegative for both PV and CB_1_. This observation might indicate that some of the boutons were false negative, i.e., we actually overestimated the number of boutons lacking PV or CB_1_. Alternatively, these boutons might originate from other GABAergic cell types, including from other amygdala nuclei (Asede et al., 2015).

Recent studies have demonstrated that PCs in the amygdala are not uniform, but differ in their afferent and efferent projections, which might endow them with defined functions (Herry et al., 2008; Senn et al., 2014; Namburi et al., 2015). These PC types are intermingled in the BLA in a salt-and-pepper manner, but may be distinctly inhibited by local interneurons (Hübner et al., 2014). These data raise a possibility that the different types of perisomatic region-targeting interneurons might provide specific patterns of GABAergic innervation onto functionally distinct amygdalar PCs, similarly to that observed in the CA1 (Lee et al., 2014). This hypothesis should be clarified in future studies.

As reported earlier in the rat hippocampus (Halasy et al., 1996) and cat visual cortex (Somogyi et al., 1983), we found that in addition to the soma and proximal dendrites, PVBCs also target the most proximal axonal segment (from soma up to 10 μm). This membrane portion of PCs, the axon hillock, might belong functionally to the soma where the expression of voltage-gated Na^+^ channels is low (Veres et al., 2014). In contrast, the more distal part of the AIS (between 15 and 60 μm), where the action potential generation most likely occurs, is targeted by AACs (Veres et al., 2014).

### Wiring features and output properties of the three interneuron types in the BLA

Our morphological analysis revealed functionally relevant differences in both dendritic and axonal arborization among the examined interneurons. For instance, BCs occupied significantly larger areas within the BLA and bore more axonal varicosities than AACs, which predict that more PCs can be innervated by single BCs than AACs (Figure 6). This is in line with observations in the neocortex (Packer and Yuste, 2011; Inan et al., 2013). Importantly, not only the number of PCs that single PV-expressing cells target is similar in neocortex and BLA, but also the fraction of the innervated PCs within a tissue slab containing the axonal field of interneurons is comparable (present study, Packer and Yuste, 2011; Inan et al., 2013). Furthermore, our results allowed us to estimate the convergence of BCs on single PCs (Figure 6). Although no such estimate for CCK/CB_1_BCs in any cortical structures has been published to date, our data for PVBCs are comparable to those revealed recently in the mouse hippocampus (14–15 in Lee et al., 2014), but differs from those calculated for neocortical networks (23 in Tamás et al., 1997, 46 in Packer and Yuste, 2011). The discrepancy among these results might be at least partially explained by the distinct methods used to estimate the convergence, and/or the neocortical networks may be distinctly wired compared to the BLA and hippocampus.

### Functional implications

A recent *in vivo* study has uncovered that in the amygdala AACs and PVBCs display markedly distinct spiking behavior during different brain states and in response to noxious stimuli (Bienvenu et al., 2012). Although there is currently no published data on how CCK/CB_1_BCs in the BLA discharge *in vivo*, data obtained in the hippocampus (Klausberger et al., 2005) imply that this third inhibitory cell type targeting perisomatic membranes could provide GABAergic inputs onto the PCs at distinct temporal windows compared to PV-expressing interneurons. Therefore, we assume that to maximize the efficacy of firing control in BLA PCs or to provide a flexible and brain state-dependent regulation of PC activities, the spiking behavior of three interneuron types should be combined or temporarily separated, respectively, depending on the functional demands of the amygdalar operation. Such synchronization or temporal segregation in firing of these interneurons can be achieved by intra- and/or extra-amygdalar afferents (Smith et al., 2000; McDonald et al., 2005), if these inputs can differentially recruit the three interneuron types. Thus, to understand the function(s) these GABAergic cells fulfill in the amygdala, future studies should reveal the properties of cortical and subcortical inputs onto these interneurons, in addition to their spiking behavior during distinct brain states.

## Author contributions

NH and JV designed the experiments. VV, JV, KM, GN, BR, and BB acquired data. VV, JV, KM, GN, and BR analyzed the data with support from NH. NH wrote the manuscript with contributions from JV and comments from all other authors.

## Funding

This work is supported by a fellowship of the Hungarian Academy of Sciences (Lendület, LP2012-23) and the National Office for Research and Technology (OMFB-01678/2009) awarded to NH. VV is supported by a European Research Council Advanced Grant (ERC-2011-ADG-294313, SERRACO). BR is supported by the Hungarian Scientific Research Fund (OTKA-K83830) and by the 9877-3/2015/FEKUT grant of the Ministry of Human Resources.

### Conflict of interest statement

The authors declare that the research was conducted in the absence of any commercial or financial relationships that could be construed as a potential conflict of interest.
